# Spatio-temporal processing of tactile stimuli in autistic children

**DOI:** 10.1038/srep05985

**Published:** 2014-08-07

**Authors:** Makoto Wada, Mayuko Suzuki, Akiko Takaki, Masutomo Miyao, Charles Spence, Kenji Kansaku

**Affiliations:** 1 Systems Neuroscience Section, Department of Rehabilitation for Brain Functions, Research Institute of National Rehabilitation Center for Persons with Disabilities, Tokorozawa 359-8555, Japan; 2 Developmental Disorders Section, Department of Rehabilitation for Brain Functions, Research Institute of National Rehabilitation Center for Persons with Disabilities, Tokorozawa 359-8555, Japan; 3 Information Center for Persons with Developmental Disabilities, National Rehabilitation Center for Persons with Disabilities, Tokorozawa 359-8555, Japan; 4 Chichibu Gakuen, Rehabilitation Services Bureau, National Rehabilitation Center for Persons with Disabilities, Tokorozawa 359-8555, Japan; 5 Division of Developmental Neuropsychology, Department of Psychological Medicine, National Center for Child Health and Development, Setagaya, Tokyo 157-8535, Japan; 6 Crossmodal Research Laboratory, Department of Experimental Psychology, Oxford University, Oxford OX1 3UD, UK; 7 Brain Science Inspired Life Support Research Center, The University of Electro-Communications, Tokyo 182-8585, Japan

## Abstract

Altered multisensory integration has been reported in autism; however, little is known concerning how the autistic brain processes spatio-temporal information concerning tactile stimuli. We report a study in which a crossed-hands illusion was investigated in autistic children. Neurotypical individuals often experience a subjective reversal of temporal order judgments when their hands are stimulated while crossed, and the illusion is known to be acquired in early childhood. However, under those conditions where the somatotopic representation is given priority over the actual spatial location of the hands, such reversals may not occur. Here, we showed that a significantly smaller illusory reversal was demonstrated in autistic children than in neurotypical children. Furthermore, in an additional experiment, the young boys who had higher Autism Spectrum Quotient (AQ) scores generally showed a smaller crossed hands deficit. These results suggest that rudimentary spatio-temporal processing of tactile stimuli exists in autistic children, and the altered processing may interfere with the development of an external frame of reference in real-life situations.

Autism is a neuropsychiatric disorder that is characterised by a severe and sustained impairment in social interaction, deviance in communication, and patterns of behaviour and interests that are restricted and/or stereotyped^1^. Although the aetiology of autism has not yet been determined, various hypotheses about its cause have been put forward^2^,^3^,^4^. For instance, Rizzolatti suggested the “mirror neuron hypothesis”, according to which, autism is caused by a disruption to the system that normally monitors one's own and others' body parts - this is referred to as the mirror system^3^,^5^. The ability to imitate gestures is also impaired in autistic children^6^, and such deficits have been explained in terms of a dysfunction of the mirror neuron system^7^. Mentalizing about the self and others (i.e., theory-of-mind) has also attracted a great deal of attention from researchers^4^. According to Baron-Cohen's “mind-blindness hypothesis”, an impairment in social interaction may be caused by deficits in the theory-of-mind. That is, the ability to attribute mental states to the self and others, and to understand that others have beliefs that may differ from one's own. According to the results of recent neuroimaging studies, individuals with autism exhibit decreased activation in frontal areas, the limbic system, and in temporal and parietal areas, including the temporo-parietal junction (TPJ) within the PPC, during a theory-of-mind task^8^. It has also been suggested that neural circuits related to the mirror system (e.g., the superior temporal sulcus) may also be involved in mentalizing about the self and others^9^,^10^.

Multisensory integration deficits may costitute a core deficit in autism, and autistic perception may be “monochannel”, meaning that attention to one sensory modality may impair an individual's ability to perceive and attend to another^11^. Previous studies investigating temporal processing in autistic children have also suggested that this population shows various unique patterns of performance when engaged in tactile temporal order judgment (TOJ) tasks involving one hand^12^, during auditory or multisensory TOJs or simultaneity judgment tasks^13^,^14^, and in the multisensory two-flash illusion task^15^.

Frith and De Vignemont^16^ have investigated the limits on mentalizing that are associated with autism. Their suggestion was that egocentric and allocentric stances interact with each other in neurotypical adults, with allocentric knowledge of others being based on, and influencing, egocentric interactions, which is the relation between self and others. These researchers also suggest that autism may involve a breakdown in this mutual dependency due to several possible cognitive deficits underpinned by the need to code information in multiple reference frames. It has also been suggested that there are multiple reference frames for spatio-temporal perception and memory in normal individuals^17^,^18^, and that these various frames of reference exist in parallel, combining to support behaviour in accordance with the specific task context^19^.

The neuronal basis for coding the various frames of reference has been investigated extensively in electrophysiological studies that have been conducted in monkeys. Neurons in the PPC have been shown to encode egocentric reference points in terms of eye-centred, head-centred, and body centred sites. For example, neurons in the lateral intraparietal sulcus (LIP; superior parts of the PPC) have a visual receptive field (RF) in an eye-centred reference frame^20^. Neurons in the LIP appear to encode the location of stimuli according to an allocentric frame of reference, whereas neurons in area 7 (the more ventral side of the PPC) encode egocentric reference frames instead^21^. Recent fMRI studies have revealed that these reference frames are primarily represented in the PPC, including the TPJ^22^.

Updating reference frames following the adoption of an unusual posture can sometimes give rise to paradoxical experiences. For example, the Japanese illusion, which is an historically well-known somatosensory-visual illusion, occurs when neurotypical participants cross their wrists, clasp their hands together with their thumbs facing downward, and then turn their hands in towards their body until their fingers face upward^23^,^24^; people typically find it difficult to move a designated (i.e., pointed to) finger correctly at the beginning of the movement, often moving one of their other digits instead^24^. Moreover, when people cross their arms over their body midline, the subjective rank ordering of successive unseen tactile stimuli delivered to both hands can be affected and is often reversed at small interstimulus intervals (this is known as the “crossed-hands effect”)^25^,^26^. The latter effect has been related to a confusion associated with the coding of stimuli in multiple reference frames when the two stimuli are presented within approximately 300 ms of each other. Given that the congenitally blind are completely unaffected by the crossing of their hands, the neural mechanisms underlying the crossed-hands effect would appear to develop during the first few years of life^27^,^28^ and the normal crossed-hands effect starts to be observed in normal children only once they have reached 5 years of age^29^. It would seem possible that autistic children may use multiple reference frames in a different manner to neurotypical individuals.

The present study was therefore designed in order to examine the spatio-temporal processing of tactile stimuli in autistic and neurotypical children using a tactile temporal order judgment (TOJ) task in which the participants adopted the crossed-hand posture. We hypothesised that autistic children would exhibit a distinctive pattern of integration of the two reference frames (e.g., somatotopic and external) when performing a tactile TOJ task while adopting a posture (crossed hands) that normally gives rise to an illusory reversal of temporal perception, which is thought to be caused by a conflict between a somatotopic and an external reference frame during the judgment process^26^. The results reported here demonstrate that the autistic children exhibit a significantly smaller crossed-hand effect than neurotypical children when their arms are crossed.

## Results

Ten autistic children (eight boys) and 10 neurotypical children (seven boys) judged the temporal order of pairs of tactile stimuli that were delivered to their ring fingers; their arms were uncrossed in one condition (arms-uncrossed condition), while they were crossed in the other (arms-crossed condition).

### TOJs with the arms-uncrossed

**Figure 1:**
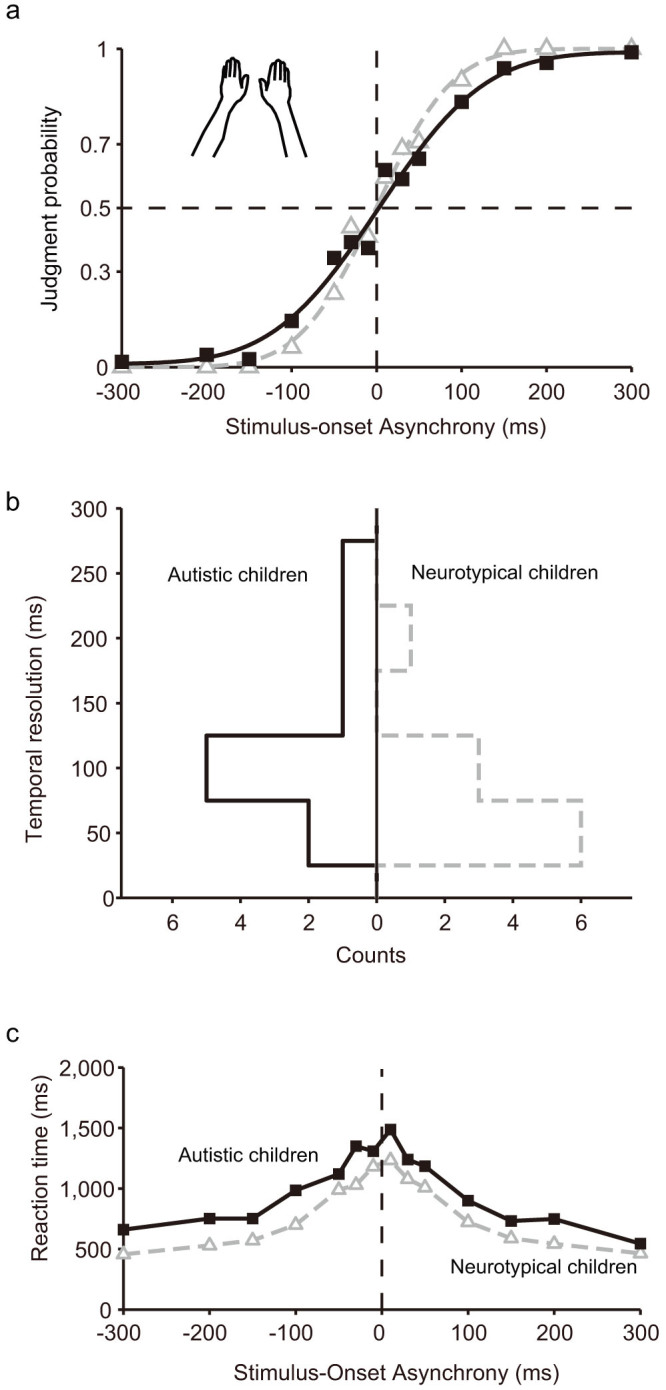
Tactile TOJs under the arms-uncrossed condition. (a) Judgment probabilities of autistic (black solid symbols) and neurotypical (gray open symbols) children. The judgment probability (ordinate) that participants reported that their left hand was stimulated second was plotted against stimulus-onset asynchrony (abscissa). The black and gray solid lines highlight the results of model-fitting^25^,^30^ for the autistic and neurotypical children, respectively. Each symbol represents the average of the 60 judgments made by the 10 autistic participants and the 60 judgments made by the 10 neurotypical participants. (b) Distributions of the temporal resolution (*σ_u_*) in autistic (black line) and neurotypical (gray line) children. (c) Reaction times (RTs) of autistic (black solid symbols) and neurotypical (gray open symbols) children under the arms-uncrossed condition. RTs (ordinate) are plotted as a function of the SOA (abscissa). The drawings of arms were drawn by W.M.

When the participants' arms were uncrossed, the order-judgment probability (*p_u_*) that the right hand was stimulated first (open circles and solid squares, Fig. 1a) was closely approximated by a monotonic sigmoid function (Eq. 1) in both the autistic and neurotypical children (solid black curve and dashed gray curve, respectively, in Fig. 1a; *r*^*2*^ = 0.99 and *r*^*2*^ = 0.99, respectively). Figure 1b shows the distribution of temporal resolution calculated for individual autistic (left histogram) and neurotypical (right histogram) children. Compared to neurotypical children (mean ± S.E. = 82 ± 15 ms, *n* = 10), autistic children (mean ± S.E. = 125 ± 21 ms, *n* = 10) have significantly lower temporal resolution (*σ_u_*) that corresponded to the stimulus-onset asynchrony (SOA) yielding approximately 84% correct responses (relative to the asymptote; *P* = 0.03 < 0.05, 2 tailed, Wilcoxon rank sum test). No significant difference was observed between the autistic and neurotypical children in the other fitting parameters in the arms-uncrossed condition (*d_u_, p_max_*, and *p_min_* in Eq. 1) (see Table 1).

We also analysed the reaction times (RTs) of each group (see Fig. 1c). A two-way ANOVA [(autistic or neurotypical) x (SOA)] revealed significant main effects of group (autistic or neurotypical; *F* = 45.8, *P* < 0.0001) and SOA (*F* = 19.4, *P* < 0.0001). No interactions were observed. The mean RT for autistic children was 989 ± 37 ms, and that of neurotypical children was 759 ± 28 ms (*P* < 0.000001, 2 tailed, Wilcoxon rank-sum test). However, no significant change was observed at any of the SOAs, which means that, in general, a slight motor-deficit was observed in the autistic children.

### Temporal order judgments in the arms-crossed condition

**Figure 2:**
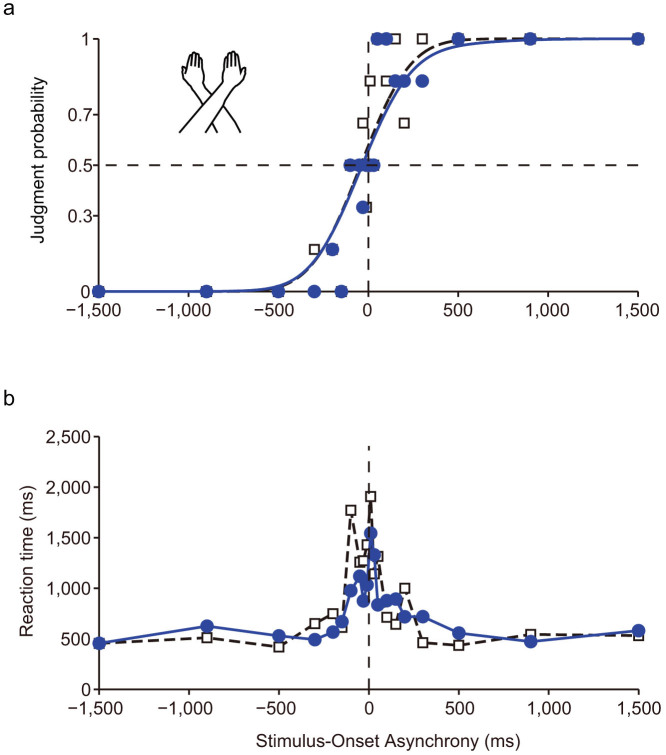
Representative data for the tactile TOJs of autistic children under the arms-uncrossed (black open squares) and arms-crossed (blue solid circles) conditions. (a) Judgment probability. (b) RTs. Notice that there is no difference between arms-uncrossed and arms-crossed conditions in this participant. Each symbol represents the average of six judgments. The drawings of arms were drawn by W.M.

After performing the tactile temporal order judgment (TOJ) task in the arms-uncrossed condition, each participant engaged in the same task in the arms-crossed condition, as in Wada et al.'s^30^ recent study. In previous research^25^,^26^,^30^, neurotypical participants revealed apparent TOJ reversals when their arms were crossed. In contrast, Figures 2a and 2b show representative responses and RTs of an autistic child (15 y.o., male) with delayed language development and an atypical gaze pattern, according to the pervasive developmental disorders autism society Japan rating scale (PARS, Spectrum Publishing Company, Tokyo). Responses in the arms-crossed and arms-uncrossed conditions were almost identical, and RTs under the two conditions were also similar.

**Figure 3:**
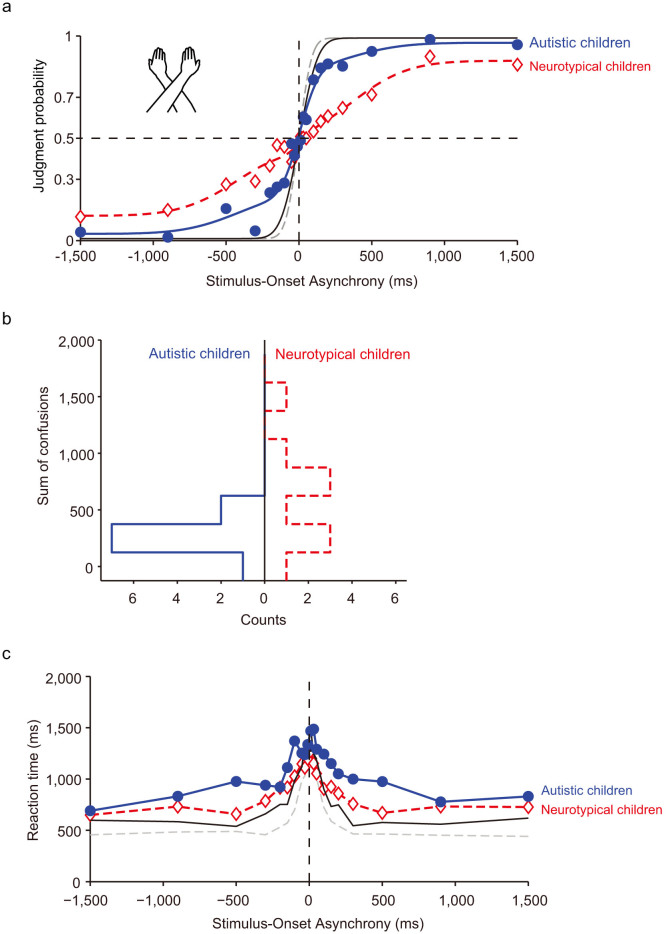
Tactile TOJs under the arms-crossed condition. (a) Judgment probabilities for autistic (blue solid circles) and neurotypical (red open diamonds) children. Judgment probabilities under the arms-uncrossed condition are also displayed (same data as in Fig. 1). Each symbol represents the average of the 60 judgments made by the 10 autistic participants and the 60 judgments made by the 10 neurotypical participants. (b) Distribution of the sums of confusion (*SC*) in autistic (blue line) and neurotypical (red line) children. Notice that the *SC*s were smaller in autistic than in neurotypical children. (c) RTs of autistic and neurotypical children. RTs under the arms-uncrossed condition are also displayed (same data as in Fig. 1). The drawings of arms were drawn by W.M.

The pooled data from all 10 participants reflected the same tendency. Although the data from the autistic children showed only a minor TOJ reversal in the arms-crossed condition, the order-judgment probability under this condition was approximated by the double-flip model^25^ of TOJ reversals (solid blue curve in Fig. 3a; *r*^*2*^ = 0.99). The model parameters (*Ã_l_* = 0.1, *Ã_r_* = 0.16, *c* = 0.02, *σ_f_* = 368 ms, *D* = −45 ms) indicate that autistic children rarely committed reversals in the TOJ task under the arms-crossed condition. In contrast, neurotypical children (red open triangles, Fig. 3a) demonstrated inverted judgments significantly more often under the arms-crossed than under the arms-uncrossed condition (dashed gray curve in Figs. 1a, 3a). The order-judgment probability in the arms-crossed condition was approximated by the double-flip model in the neurotypical children (dashed red curve in Fig. 3a; *r*^*2*^ = 0.97). The model parameters (*Ã_l_* = 0.31, *Ã_r_* = 0.27, *c* = 0.12, *σ_f_* = 386 ms, *D* = −32 ms) were quite similar to those in previous reports involving adult participants^25^,^30^.

The model parameters (*Ã_l_*, *Ã_r_*, *c*, *σ_f_*, and *D*) from the functions were calculated for each participant (see Table 1). The net peak flip at R-L stimuli (*Ã_r_*) of autistic children were significantly lower than were those of the neurotypical children (*P* = 0.006 < 0.01, 2 tailed, Wilcoxon rank sum test, see Table 1). Additionally, the net peak flip at L-R stimuli (*Ã_l_*) was also significantly smaller in autistic children (*P* = 0.04 < 0.05, 2 tailed, Wilcoxon rank sum test, Table 1). The net peak flips (Ãr, Ãl) can be used to provide an indication concerning the degree of the TOJ reversals (“right first”, “left first”). The two groups did not differ significantly with regard to the other parameters (c, *σ_f_* and *D*). Moreover, the flip probabilities were so large in 5 out of the 10 neurotypical children that the response curves were no longer sigmoidal, but became N-shaped; this response curve was not observed in the autistic children.

To examine the degree of TOJ reversals, we evaluated reversals or confusions in terms of the sum of confusions (*SC*) (eq. (7)) and the proportion-correct difference (*PCD*^31^,). The *SC* and *PCD* provide a rough metric indicating the increase in reversals between the two response functions that result from arm-crossing. Figure 3b shows the distribution of the *SC* calculated for individual autistic (left histogram) and neurotypical (right histogram) children. The *SC* was significantly smaller (*P* = 0.04 < 0.05, 2 tailed, Wilcoxon rank sum test) in the autistic children (mean ± S.E. = 260 ± 42, *n* = 10) than in the neurotypical children (mean ± S.E. = 654 ± 143, *n* = 10). We also calculated the PCD score, which provides a rough indication of increases in TOJ reversals and, unlike fitting functions, does not require any assumptions^31^. PCD scores were also significantly lower (*p* = 0.0091 < 0.01, 2 tailed, Wilcoxon rank sum test) in the autistic (mean ± S.E. = 1.67 ± 0.39, *n* = 10) than in the neurotypical (mean ± S.E. = 4.72 ± 0.65, *n* = 10) children (see Table 1). These results suggest that spatial confusion due to arm-crossing was less common in the autistic children than in the neurotypical children.

**Figure 4:**
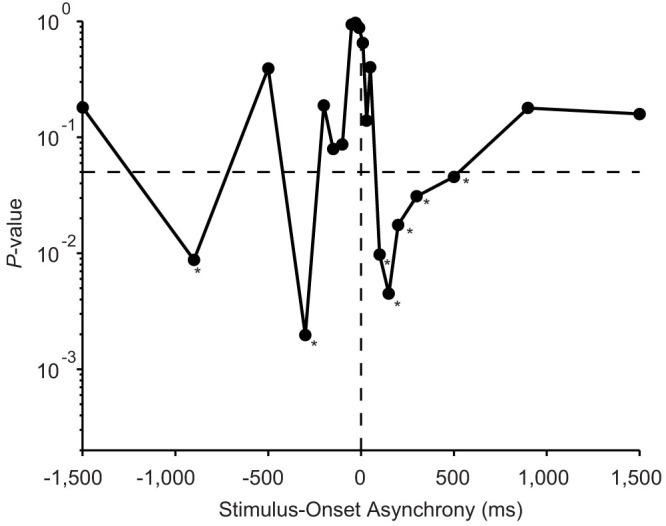
Difference between autistic (*n* = 10) and neurotypical (*n* = 10) children in the judgment probability at each SOA under the arms-crossed condition. The *P*-value (ordinate) is plotted as a function of the SOA (abscissa).

We further analysed the judgment probabilities of each group in the arms-crossed condition. A two-way ANOVA [(autistic or neurotypical) x (SOA)] revealed significant main effects of SOA (*F* = 31.8, *P* < 0.0001) and significant interactions between group and SOA (*F* = 3.1, *P* < 0.0001). We compared judgment probabilities between the groups at each SOA in order to clarify the critical range of stimulation intervals. Significant differences between the autistic and neurotypical children were observed at particular SOAs (in the 100–300 ms range) when responses (judgment probability) were compared under the arms-crossed condition at each SOA (Fig. 4, *P* < 0.05, Wilcoxon rank sum test). This means that the difference between the responses of autistic and neurotypical children reached its maximum at around the particular SOA (100–300 ms) at which the flips peaked in the neurotypical participants^25^,^30^. This result suggests that TOJ reversals due to arm-crossing were actually less prevalent in the autistic group. Such decreases in TOJ reversals suggest that somatotopic cues take priority over spatial cues when autistic children perform the TOJ task.

Following the procedure used for the arms-uncrossed condition, the RTs of each group under the arms-crossed condition were analysed (see Fig. 3c). A two-way ANOVA [(autistic or neurotypical) x (SOA)] revealed significant main effects of group (autistic or neurotypical; *F* = 15.7, *P* = 0.0001< 0.001) and SOA (*F* = 6.0, *P* < 0.0001). No interactions were observed. The mean RT of the autistic children was 1058 ± 33 ms, while that of the neurotypical children was 907 ± 26 ms (*P* = 0.0015 < 0.01, 2 tailed, Wilcoxon rank sum test).

### Response in the unilateral stimulation trials

During the experiment, two successive stimuli delivered to the same ring finger (L-L and R-R; unilateral stimulation trials) were randomly intermixed as a control condition. In the unilateral stimulation trials, the participants were required to press a button with the index finger of the stimulated hand following the delivery of the stimuli.

**Figure 5:**
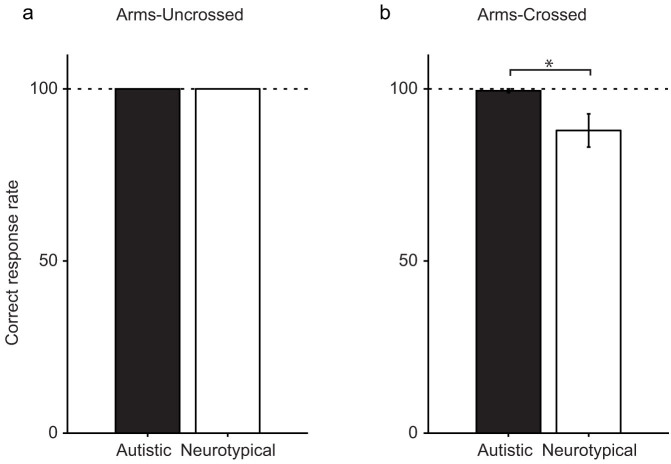
Correct response rate of the unilateral stimulation trials in the arms-uncrossed condition (a) and arms-crossed condition (b).

In the arms-uncrossed condition, the participants in both groups responded with an accuracy of 100% (Fig. 5a). In contrast, the autistic group exhibited more precise judgments than did the neurotypical children (*P* = 0.015 < 0.05, 2 tailed, Wilcoxon rank sum test) in the arms-crossed condition, although most participants responded correctly in most of the trials (autistic and neurotypical children: 99.4 ± 5.6 and 87.9 ± 3.9%, respectively, Fig. 5b). This result is also consistent with the significant decrease in spatial confusion observed amongst the autistic children during arm-crossing.

### Relationship between AQ scores and the degree of the crossed hand illusion

**Figure 6:**
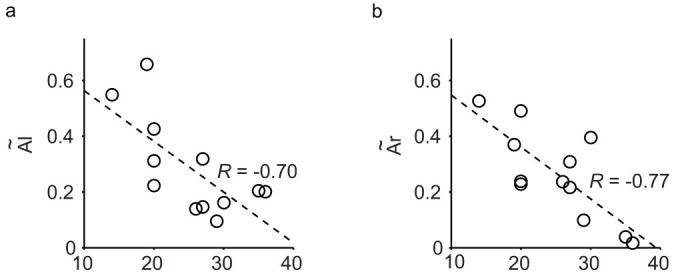
Relationship between Japanese version of Autism Spectrum Quotient (AQ) scores and the degree of the crossed hand illusion (*n* = 12). The fitting parameters (*Ã*_l_ (a) and *Ã*_r_ (b); ordinate) are plotted against the AQ score (abscissa) of individual participants.

We recruited further 12 young boys, who join a self-help group for mild developmental disorders and who also attended a regular class at school, and whose Autism Spectrum Quotient (AQ) scores ranged from 14 to 36 (mean: 24.5 ± 2.0). They were asked to perform the TOJ tasks, and we evaluated the relationship between their AQ scores and the magnitude of the crossed hand illusion. The order-judgment probability (*p_u_* and *p_c_*) were also closely approximated by same functions (Eq. 1 and 2), and large individual differences in the magnitude of the crossed hand illusion were observed. In particular, both left-to-right flip probabilities (*Ã*_l_) and right-to-left flip probabilities (*Ã*_r_) were negatively correlated with the AQ score (R = −0.70, *P* = 0.001 < 0.05; R = −0.77, *P* = 0.035 < 0.01) (Fig. 6a, b, respectively). None of the other parameters (*t, d_u_, σ_u_, p_max_*, *p_min_, c, D*, and *σ_f_*) in either the arms-uncrossed or arms-crossed conditions were correlated with the AQ scores. The young boys who had a higher AQ score generally exhibited a smaller crossed hands illusion especially at moderately short intervals consistent with the results with children with autism (Figs. 2–4).

## Discussion

The present study investigated how the autistic brain processes spatio-temporal information concerning tactile stimuli. Children with autism performed a crossed-hands-illusion task that often elicits a subjective reversal of TOJs among neurotypical individuals when both hands are stimulated in close succession while crossed. Our results demonstrate that the reversal illusion was significantly less prominent in autistic than in neurotypical children.

Updating reference frames sometimes gives rise to paradoxical experiences, and previous studies have suggested that the conversion of reference frames may be a source of the illusion during the TOJ task with crossed hands^25^,^26^,^28^,^30^,^31^,^32^. Yamamoto and Kitazawa^25^ have previously suggested that the brain orders events in time, after cutaneous signals have been localised in space, by factoring in the positions of the hands. Azañón and Soto-Faraco^33^, meanwhile, have suggested that a fleeting, unconscious image of the tactile sensation coded according to a somatotopic frame of reference governs performance prior to tactile events being referred to external locations. Shore et al.^26^ suggested that the reversal illusion during a TOJ task with crossed hands may reflect a conflict between a somatotopic and an external reference frame during the judgment process.

Spatial memories, which are closely related to reference frames, are considered to be supported by multiple parallel representations, including somatotopic and external ones; these are updated to accommodate self-motion^19^. In neurotypical individuals, somatotopic (intrinsic) cues from the primary somatosensory cortex are converted into an external frame of reference in real-life situations. Several neuroimaging studies that have involved the performance of spatial tasks have indicated that such multiple reference frames are represented in the posterior parietal cortex (PPC), including the TPJ^17^,^22^. Previous studies have also suggested that the PPC is important for switching between first- and third-person viewpoints^34^. For example, it has been reported that electrical stimulation of the TPJ can give rise to out-of-body experiences^35^, and activity in the PPC increases under conditions involving higher levels of self-identification (e.g., first- vs. third-person perspective)^34^.

In contrast, individuals with autism are thought to experience a disconnection between a strong egocentric stance and a highly abstract allocentric stance^16^. Clinical evidence suggests that the multiple-reference-frames system may not work well in children with autism. For example, doctors treating and recruiting these children with autism have reported that they frequently wave backwards during childhood. Psychological experiments conducted on children with autism have demonstrated that such palm-reversal errors are also observed during imitation tasks and it has been suggested that these phenomena may be caused by the inability to integrate body parts into a whole based on visual input^6^.

The reference frame in which the body parts are coded is hypothesized to be computationally represented in the cerebellum, and the internal model found in the autistic brain in this region may simply be different from that seen in the neurotypical brain. Shadmehr and colleagues measured patterns of generalization as children learned to control a novel tool and found that children with autism weight proprioception (intrinsic coordinates) more in perceptual inference^36^. Moreover, they reported that the greater the reliance on proprioception, the greater the child's impairments in social function and imitation. The results of the present study demonstrate that the reversal illusion was significantly less pronounced in autistic than in neurotypical children, suggesting that the implicit processes underpinning an abstract allocentric stance may be impaired in the former^16^,^37^. As a possible explanation, the impairment may be caused by the dysfunction of the allocantric stance itself, or by a distinctive integration of the egocentric and allocentric frames of reference that makes it difficult to isolate the allocentric coding independent of the egocentric coding.

Space and time are sometimes related and can interact in human information processing^38^,^39^. Shore et al. noted that a conflict between a somatotopic and an external reference frame can be highlighted by examining the marked deficit related to tactile TOJs when the hands are crossed^26^,^31^. The present study demonstrates that the reversal illusion is significantly smaller in autistic children than in neurotypical children while adopting this, somewhat unusual, posture.

Recent advances in neuroimaging have allowed researchers to investigate the neural bases for such behaviours, and studies investigating the neural representation of the reference frame during spatial tasks have emphasised the importance of the right PPC^17^,^22^,^40^. In contrast, when temporal information is involved (e.g., movement, successive stimuli, and language), activity is predominantly observed in the left PPC^41^,^42^,^43^,^44^,^45^. Takahashi et al.^46^ had their participants perform a tactile TOJ task while lying in the fMRI scanner and observed the bilateral activation of the PPC, and further reported left dominant activation in the PPC when their participants' arms were crossed as compared to when they were uncrossed. In a previous study, we demonstrated increased fMRI activation in the left PPC when human participants adopted a crossed-hands posture^32^. We also observed a positive association between fMRI activation in the left PPC, especially the intraparietal sulcus, and the degree of TOJ reversals resulting from arm crossing. This result implies that the left PPC may be critically involved in monitoring limb position and in spatio-temporal binding when serial stimuli are delivered to the limbs. These multisensory brain areas may, then, be a key to further understanding the neuronal basis of autism in children.

Canonical representations of the body, including the hands, are multisensory^47^. Röder et al.^28^ indicated that congenitally blind individuals experience superior temporal resolution during tactile TOJ tasks when their arms are uncrossed and do not show clear TOJ deficits due to arm-crossing. They also indicated that congenitally blind individuals appear to use an anatomical reference frame (somatotopic representation of touch) for the multisensory control of action^48^. Their research also suggested that the critical role of childhood vision in modulating tactile perception may arise from the emergence of specific crossmodal links during development. They also observed an obvious crossed-hand effect in neurotypical children older than 5 year of age^29^. Such results suggest that the system of multiple reference frames is characterised by multisensory input, and that the system may develop during the first few years of life. Impairments in multisensory integration may be a core deficit in autism, and autistic perception may be “monochannel”, meaning that attention to one sensory modality may impair perception and attention via another^11^. Consistent with the “monochannel” hypothesis, the rubber hand illusion (RHI), which is characterized by illusory body image and cross-modal interactions between vision and touch^49^,^50^,^51^, has been shown to be relatively weak in autistic participants^52^,^53^. The results of the present study therefore suggest that such crossmodal links may be weak in autistic children, as well as in those individuals who are congenitally blind^28^.

Previous studies investigating temporal processing in autistic children have suggested that this population shows various unique patterns of performance when engaged in tactile TOJ tasks involving one hand^12^, during auditory or multisensory TOJ or simultaneity judgment tasks^13^,^14^, and in the multisensory two-flash illusion task^15^. The distinctive patterns of integration of somatotopic and external reference frames in autistic children observed in the current study may involve issues related to spatio-temporal information processing. The lower temporal resolution in the arms-uncrossed condition and general slower RTs observed in the current study might reflect the possibility that there is a general sensory-motor integration deficit in autistic children. In contrast, a smaller crossed-hand illusion and a higher overall correct response rate in the arms-crossed condition indicates that unique and rudimentary spatio-temporal information processing of tactile stimuli exists in the autistic children.

It has been established that an individual's susceptibility to TOJ reversals is subject to substantial inter-individual differences^25^. For instance, Cadieux et al.^31^ reported inter-participant variability in the confusion experienced by neurotypical volunteers in the tactile TOJ task as a result of arm-crossing and indicated that arm-crossing is generally more confusing for females than for males. According to their results, three of 24 male participants actually demonstrated almost no confusion under the arms-crossed condition, whereas this was not true for any of the female participants. Our results demonstrate smaller TOJ reversals in autistic children. In our experiment, the young boys with higher AQ scores generally showed a smaller crossed hands illusion. Furthermore, the AQ scores were higher in males (mean: 18) than females (mean: 15)^54^. These results are consistent with the fact that autism is more common in males than in females. The “extreme male theory of autism” may provide useful information to further clarify the point^55^,^56^,^57^.

In summary, the results of the present study demonstrate that the reversal illusion was significantly less prominent in autistic children than in neurotypical children. Furthermore, young boys who have higher Autism Spectrum Quotient (AQ) scores generally show a smaller crossed hands illusion. The results imply that rudimentary spatio-temporal processing of tactile stimuli exists in autistic children, and the altered processing may interfere with the development of an external frame of reference in real-life situations.

## Methods

### Participants

The study was approved by the institutional ethics committee at the National Rehabilitation Center for Persons with Disabilities, and all of the participants and their parents provided written informed consent according to institutional guidelines. Twenty autistic and neurotypical children participated in the experiment in total. The autistic group consisted of 10 children ranging from 9 to 15 years of age (eight boys and two girls; 11.8 ± 0.7 y.o). These autistic children had been diagnosed with pervasive developmental disorder-not otherwise specified (PDD-NOS) or high-functioning autism according to the DSM-IV (American Psychiatric Association 2000). Diagnoses were established based on the clinical judgement of two medical specialists. According to DSM-V (American Psychiatric Association 2013), individuals with a well-established DSM-IV diagnosis of autistic disorder, Asperger's disorder, or PDD-NOS should be given the diagnosis of autism spectrum disorder. Thus, the participants were diagnosed as autism spectrum disorder. All of the autistic children had normal IQs (78–136, 100.7 ± 6.5) according to the Wechsler Intelligence Scale for Children-Third Edition (WISC-III; The Psychological Corp., San Antonio, TX). Eight of the autistic participants were judged to be strongly right-handed (+50 ≤ L.Q. ≤ +100) according to the Edinburgh Inventory^58^. The other two children were classified as left-handed (LQ = −20, −50). Three additional children were tested but excluded from the analysis due to the fact that they could not remain awake or keep their eyes closed during the task. Ten neurotypical children (7 boys and three girls; 11.7 ± 0.7 y.o) who have normal IQs (89–112, 101.6 ± 2.4) without family histories of autism also took part in the study. Nine participants were judged to be right-handers (+50 ≤ L.Q. ≤ +100). The other child was ambidextrous (LQ = +30). The autistic and neurotypical groups did not differ significantly in the ratio of right-handers (Wilcoxon rank-sum test). Note here that a previous study had reported there to be no significant difference between right-handers and left-handers as to magnitude of the crossed hand illusion^30^.

Twelve young boys (16.3 ± 0.4 y.o.) who joined a self-help group for mild developmental disorders and also attended a regular school class additionally participated in the same experiment. All of the boys had normal IQs (74–107), and eleven out of the twelve were judged to be strongly right-handed (+50 ≤ L.Q. ≤ +100) according to the Edinburgh Inventory^58^. The other child was classified as left-handed (LQ = −50). In addition, they answered questionnaires on a Japanese version of Autism Spectrum Quotient (AQ) scores^59^.

### Procedure

The participants were required to sit and place their hands in a palm-down position on the top of a desk. In one condition, the participant's arms were uncrossed (arms-uncrossed condition); in the other condition, they were crossed (arms-crossed condition). In the latter condition, the participant's left arm was placed over their right arm, and the arms touched each other at the distal end of the forearm.

Solenoid skin contactors (Uchida Denshi, Tokyo, Japan) were used to deliver brief tactile stimuli (10 ms in duration) to the dorsal surface of the ring finger of each hand. The distance between the participant's ring fingers was 20 cm in all of the conditions. A small button was placed at the tip of the index finger to detect the participant's responses in each trial. During the course of the experiment, the participants were instructed to close their eyes while white noise (80 dB) was played over headphones. The participants also wore earplugs. Two successive stimuli were delivered, one to each ring finger; the stimuli were separated by one of 20 randomly assigned intervals (−1,500, −900, −500, −300, −200, −150, −100, −50, −30, −10, 10, …, 1,500 ms). Positive values indicate that the participant's right hand was stimulated first, and negative values indicate the reverse (R-L and L-R, respectively; bilateral stimulation trials). In addition to the 20 intervals, two successive stimuli were occasionally delivered to the same ring finger separated by ±1,500 ms and were randomly intermixed as a control condition (L-L and R-R; unilateral stimulation trials). The participants were instructed to press a button with the index finger of the hand that received the second stimulus. Each session included six epochs. In each epoch, the 20 + 2 intervals were presented in a random order. Consequently, one session consisted of 132 trials. The participants were encouraged to respond as rapidly as possible following the delivery of the second stimulus. Whenever the RT was either below 100 ms or exceeded 6,000 ms, a trial with the same stimulus-onset asynchrony (SOA) was presented at the end of the experiment. No feedback was given to any of the participants about the speed or accuracy of their responses. The intervals between different trials were randomly selected to fall between 1,500 and 2,500 ms.

### Data analysis

We used the same functions as described by Wada et al.^30^ in order to fit the order-judgment probabilities that the participant's right hand was stimulated earlier (or the left hand was stimulated later) in the uncrossed (*p_u_*) and crossed (*p_c_*) conditions.

First, the order-judgment probability under the uncrossed condition (*p_u_*) was fitted by a cumulative density function of a Gaussian distribution as follows: 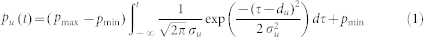
Here, *t, d_u_, σ_u_, p_max_*, and *p_min_* denote the stimulation interval, size of the horizontal transition, temporal resolution, and upper and lower asymptotes of the judgment probability, respectively.

Second, the order-judgment probability in the arms-crossed condition (*p_c_*) was fitted by the double-flip model^25^, which was flipped (i.e., reversed) from the order-judgment probability under the uncrossed condition (*p_u_*) as follows: 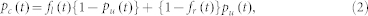


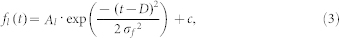


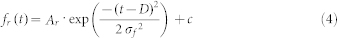
In this context, *f_l_* denotes the flip probability of judging “left first” to “right first”, and *f_r_* denotes the flip probability of judging “right first” to “left first”. Following Yamamoto and Kitazawa^25^, we estimated the five parameters in the flip probabilities that followed the Gaussian functions shown in Equations 3 and 4: the peak flip amplitudes of the Gaussian functions (*A_l_* and *A_r_*), the size of the horizontal transition (*D*), the time window of the flip (*σ_f_*), and a constant error rate (*c*). The *c* corresponds to the probability of a generic error in the responses, whereas *A_l_* and *A_r_* reflect a tendency to make judgment reversals at short inter-stimulation intervals that subsided at longer intervals. Following Wada et al.^30^, we calculated the net peak flip amplitudes (*Ã_l_* and *Ã_r_*), which were defined as follows. 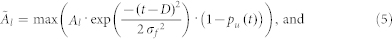


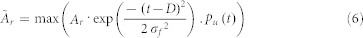
Matlab (MathWorks, Natick, MA) was used for estimations to minimise Pearson's *chi* statistics.

To evaluate increases in the reversals caused by arm-crossing, the sum of the differences between the response functions under the arms-uncrossed (*p_u_*) and arms-crossed (*p_c_*) conditions were calculated for each participant (−1500 ms ≤ *t* ≤ 1500 ms) and defined the sum as the sum of confusions (*SC*) in Equation 7. 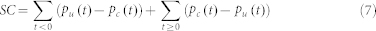
The *SC* provides a rough index of the increase in reversals resulting from arm-crossing.

We also calculated the proportion-correct difference (*PCD*) by taking the difference, at each SOA, between the performance in the crossed and in the uncrossed condition and summing these differences^31^. The *PCD* also provides a rough indicator of any increase in the likelihood of reversals resulting from arm-crossing without the need to fit any models.

## Author Contributions

W.M. and K.K. designed research; W.M., M.S., A.T., M.M. and K.K. performed research; W.M. and K.K. analyzed data; W.M., C.S. and K.K. wrote the paper.

## Figures and Tables

**Table 1:** Fitting parameters and reversal indicators for autistic and neurotypical children

	Autistic children	Neurotypical children	*P*-value
**Sex (M:F)**	8:2	7:3	n.s.
**Age**	11.8 ± 0.7	11.7 ± 0.7	n.s.
**IQ**	100.7 ± 6.5	101.6 ± 2.4	n.s.
**Uncrossed**			
σu(ms)	125 ± 21	82 ± 15	0.03*
du(ms)	4.1 ± 9.3	−6.2 ± 10.5	n.s.
Pmax	0.998 ± 0.001	0.998 ± 0.001	n.s.
Pmin	0.003 ± 0.002	0.002 ± 0.001	n.s.
**Crossed**			
*Ã*_r_	0.14 ± 0.030	0.33 ± 0.047	0.006**
*Ã*_l_	0.17 ± 0.040	0.30 ± 0.046	0.04*
C	0.048 ± 0.012	0.12 ± 0.033	0.09
σf(ms)	359 ± 50	384 ± 73	n.s.
D(ms)	76 ± 74	75 ± 42	n.s.
SC	260 ± 42	644 ± 141	0.04*
PCD	1.67 ± 0.39	4.72 ± 0.65	0.009**

Data are means ± S.E.s for 10 autistic and neurotypical children.

See Equations (1)–(7) for definitions of the parameters.
